# Current status and frontier tracking of the China HACCP system

**DOI:** 10.3389/fnut.2023.1072981

**Published:** 2023-03-14

**Authors:** Xiaoliang Shi, Xinyue Zhang, Runa A, Tielong Wang, Jiayi Zhang, Yuanpeng Liang

**Affiliations:** ^1^College of Economics and Management, Shenyang Agricultural University, Shenyang, China; ^2^School of Finance, University of International Business and Economics, Beijing, China; ^3^Chinese Academy of Inspection and Quarantine, Beijing, China

**Keywords:** HACCP, food safety, process management, control and prevention, supervision and management, CNKI

## Abstract

In today’s booming society and in the age of science and technology, the diversification of food processing methods, the continuous extension of the food trade chain, and the potential hazard factors in the food production process all make people pay more and more attention to the establishment, development, and improvement of the hazard analysis and critical control points (HACCP) system. Only terminal control and post-processing supervision of food can guarantee the absolute safety of food. In the process of processing, it is particularly important to strictly identify and evaluate the food safety hazards. To better assist food production enterprises in establishing and implementing HACCP systems, to implement the primary responsibility of food safety, and to improve the theoretical level and practical application of HACCP system in China, an investigation of the current situation and development frontier of HACCP system in China was conducted. Based on the core journal database of China Knowledge Network, the Chinese Social Science Citation Index database, and the Chinese Science Citation Database as the literature search database platform, the study used the CiteSpace visual metrics software system to analyze 1,084 pieces of literature in the field of HACCP research, in order to track the dynamics and impact of research in this field by Chinese research institutions and major authors, and analyze the research hotspots in the field. It is important for further research on HACCP. The results of the study showed that (1) the number of publications in the field of HACCP in China increased steadily from 1992 to 2004 and then began to decrease; (2) the indexes of journals with more publications were more concentrated, and the journal Food Science published the most; (3) the indexes of major research institutions showed that the cultivation bases of the State Key Laboratory of Chinese Medicinal Materials in the Center of Chinese Medicine Resources of the Chinese Academy of Traditional Medicine, the Guangdong Institute of Occupational Diseases, the Nanchang University of Life Sciences, and the Guangdong Institute of Occupational Diseases were more concentrated. Prevention and Treatment Institute, School of Life Sciences of Nanchang University, China Aquatic Products Quality Certification Center, School of Food Science and Nutrition Engineering of China Agricultural University, and other research structures have the most publications and strong scientific research strength; (4) from the main author indicators, the research in the field of HACCP has formed a total of four more active research teams, involving Chinese herbal medicine, ecological planting, ecological agriculture, occupational disease prevention and treatment, light industry handicrafts, computer software and computer application, agricultural economy, and other research directions. The cooperation between the authors of each team is closer. It is suggested that in terms of food safety requirements, China should not only integrate the traditional supervision measures for food terminals and after the event but also reflect the role of food hazard analysis and assessment in the production process and comprehensively integrate the pre-production, production, and post-production management of food so that food can really be safe.

## 1. Introduction

**Figure 1:**
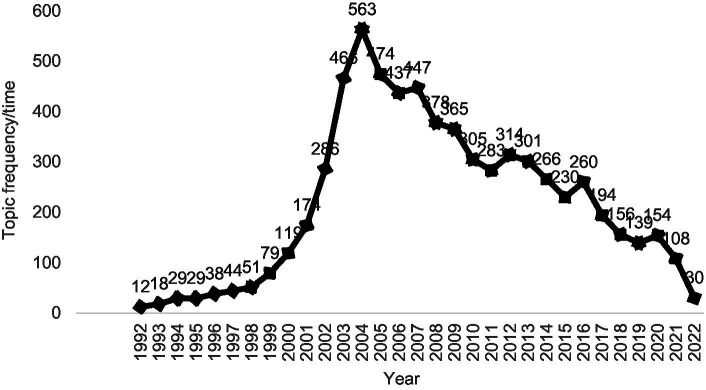
Research attention in the field of hazard analysis and critical control points (HACCP) from 1992 to 2022.

Modern industrialization and improving living standards have continuously increased people’s expectations for food safety as compared to the past (1–3). The food production industry also paying greater attention to concepts like food safety control, prevention, and process management. In recent years, it has become widely recognized and accepted that the hazard analysis and critical control points (HACCP) system is capable of ensuring the safety of food (4–7). It is important not only to meet human needs, but also to improve the standards of food safety in social enterprises. It was determined that the search period was 1992–2022, and the source database was used for the search scope. A variety of databases were used to analyze the attention given to HACCP research, including journal databases, doctoral thesis databases, master’s thesis databases, newspaper databases, and conference databases, as shown in Figure 1.

From Figure 1, it is evident that the attention of HACCP research has been on the rise since 1992, reaching its peak in 2004 with 563 research topics appearing during the year. During the period 1992 through 1997, China just stepped into the introduction phase of the HACCP system and received scant attention. In 1997, China sent a five-member expert group to participate in the first HACCP management teacher training course held by the food and drug administration (FDA) of the United States. Chinese HACCP research has been launched as a result of this study (8). In the period following 2000, researchers have been paying increasing attention to HACCP research. In 2001, China’s first HACCP certification body “China Commodity Inspection Corporation HACCP Certification Coordination Center” was established. As of 2002, China’s first special HACCP administrative rules were issued titled “Food production Enterprise Hazard Analysis and Critical Control Point (HACCP) Management System Certification Management Provisions.” During a period of time in 2004, the Certification and Accreditation Administration of China compiled six textbooks about the HACCP system, held 37 training courses, and guided more than 4,000 export food production enterprises in six categories, who all established and implemented the HACCP system, thus igniting the interest of HACCP research. After 2004, however, the attention of HACCP research slowly subsided, with minor fluctuations in the process, which has been attributed to China’s release of many standards related to food safety management systems. This reduction in attention may also be explained by the fact that the HACCP system gradually developed into a stable system.

Based on the China national knowledge infrastructure (CNKI) database as the data source, bibliometric analysis methods and literature visualization tools will be used to conduct the study. This study presents the research status, research hotspots, and development trends of China’s HACCP system using a knowledge graph, in order to serve as a useful reference for subsequent research in this field. The bibliometric analysis method was chosen because it has been widely used in quantitative literature research in a particular subject area (9–14) and CiteSpace software is also an influential visual image analysis tool that can effectively provide a comprehensive assessment of a discipline or research area (15–18). In addition, knowledge graphs can combine information visualization technology with traditional bibliometric analysis, and generate different types of knowledge graphs by integrating data mining, information processing, scientific measurement, and graph rendering to make the information more intuitive to researchers (19–23). This paper uses the methods of keyword clustering, keyword emergence and keyword periodic change of CiteSpace software to analyze and reveal the research hotspots, development ideas and strategic considerations in the field of HACCP in the future. The research results can not only improve the GMP, SSOP, GAP, and other food safety management systems but also empirically promote the “China HACCP system” to the world.

## 2. Materials and methods

### 2.1. Data sources

To ensure the authoritativeness and typicality of the research results, the following three databases in the CNKI were used: the Chinese social sciences citation index (CSSCI) Database, the Chinese science citation database (CSCD), and Peking University’s core database. The scope of the selected and analyzed literature was limited to literature written in Chinese or published in China, resulting in the absence of research papers written by Chinese scholars but published in international journal platforms, which may have some impact on the rigor of the arguments and the importance of the findings, but the study still has some significance. First of all, the CSSCI, CSCD, and Peking University (PKU) core databases are important indicators of whether Chinese scholars, research institutions, and projects have completed their research. Further, although research papers written by Chinese scholars are published in international journals, similar “shadows” can also be found in domestic journals, which will not have a significant impact on the findings and conclusions of this study. As a result of the above two points, the significance and importance of this study will not be diminished.

In the study, “HACCP” was selected as the theme of accurate retrieval, and the retrieval period covered the period was 1992–2022. By conducting a preliminary search, 1,271 articles were obtained in total. Based on this, an in-depth manual interpretation of the titles and abstracts of the literature was conducted, to remove irrelevant literature, conference announcements, and news publicity, and a total of 1,084 books were retained in this study. As soon as the retrieved literature data was exported in Refworks format and saved to plain text files, CiteSpace software was used to convert and process the inherent data, thereby obtaining relevant data that can be considered as part of the data samples that were analyzed in this study. The paper conducted an accurate search after selecting a topic to avoid the possibility of a large number of irrelevant results. After the final determination of the sample size, the author exported and transcoded the target literature according to the reference format required by CiteSpace to obtain the research sample database for this paper.

### 2.2. Methods

#### 2.2.1. CiteSpace software

CiteSpace is one of the most influential tools in bibliometric analysis (24–27). It is a Java application that analyzes and visualizes literature and is developed by Chen Chaomei, a Chinese scholar at Drexel University. It is possible to download CiteSpace software for free from the website linked to it.^1^ It is the purpose of this paper to provide a comprehensive analysis of the research status and the latest advancements in the field of HACCP in China, based on the use of CiteSpace 5.8R3C version, in addition to the application of Excel.

In CiteSpace software, the analysis of collaboration networks (main authors and research institutions), co-occurrence networks (keywords), and other statistical analysis functions provides scholars with an objective assessment of the current state of the target research field in terms of time, research institutions, and members, as well as keywords (28–30). This paper adjusts and sets the parameters of CiteSpace software according to the requirements of these functions, based on existing research practices.

#### 2.2.2. Parameter configuration

1. Time slicing was 1992–2022, and the year of each slice (Years Per Slice) = 1.
2. Node Types were, respectively, selected as research institution, author, and keyword.
3. Pruning Settings were selected as “Pruning sliced Networks”.

## 3. Results

### 3.1. Examine the fundamental characteristics of the literature

The analysis of basic characteristics of literature primarily focuses on the quantity of published articles and the main sources of the journals. Among them, a change in the number of publications is an important indicator of measuring research within a specific field (31–33). Journals that mainly provide relevant literature in a certain field can be used to assess the authoritative journals in which researchers in that field are more likely to publish research.

#### 3.1.1. Publication count

**Figure 2:**
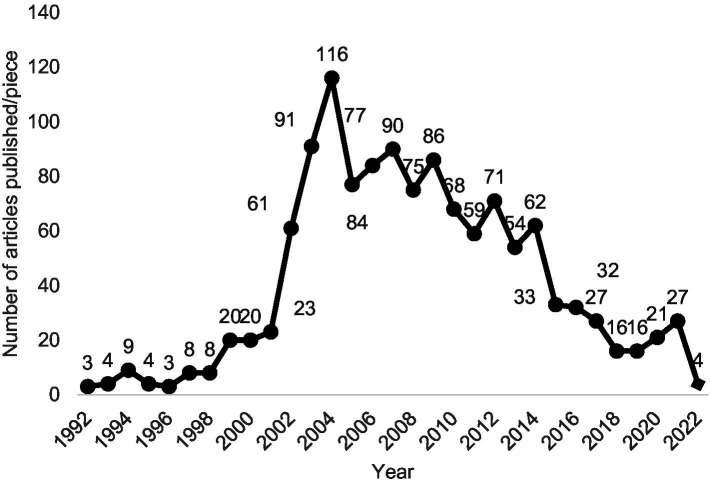
Quantity of publications in the field of hazard analysis and critical control points (HACCP).

Based on the number of papers published in the field of HACCP, the overall data can be split into two stages: 1992–2004 has an upward trend, and 2004–2022 has a downward trend, as shown in Figure 2.

As can be seen in Figure 2, there was an upward trend in the number of published papers within the field of HACCP since 2002, with the number of published papers surpassing 60 for the first time in the past few years. In 2004, the number of articles reached 116, which marked the end of this upward trend. As of 2004, the number of published articles fluctuated up and down, but on the whole, the number of publications tended to decrease.

#### 3.1.2. Primary journal sources

**Figure 3:**
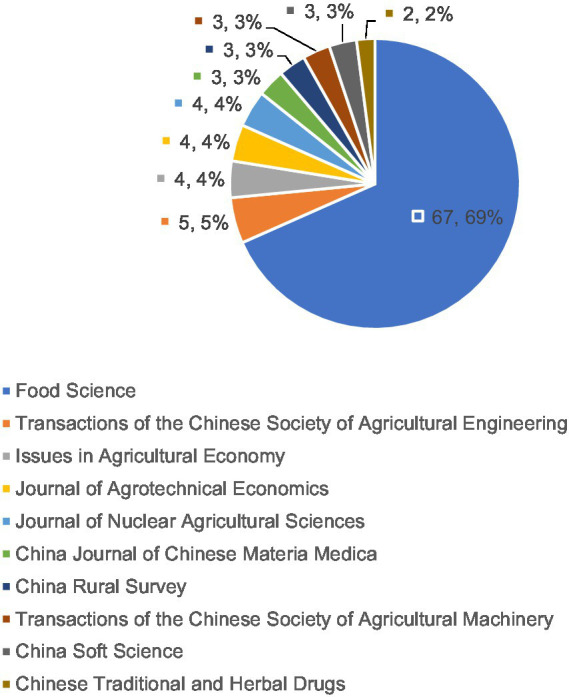
Main source journals in the field of hazard analysis and critical control points (HACCP).

It can be seen in Figure 3 that the journals selected for publication are relatively concentrated in the field of HACCP research.

In the area of HACCP, Figure 3 showed that the top 10 journals were: “Food Science, ““Transactions of the Chinese Society of Agricultural Engineering,” “Issues in Agricultural Economy,” “Journal of Agrotechnical Economics, ““Journal of Nuclear Agricultural Science,” “China Journal of Chinese Materia Medica,” “China Rural Survey,” “Transactions of the Chinese Society of Agricultural Machinery,” “China Soft Science,” and “Chinese Traditional and Herbal Drugs.” The largest number of articles were published in Food Science, accounting for 69% of the total number of articles published, with 67 articles in total. The number of articles published in other journals was relatively small, accounting for approximately 5% of the total. In light of this data, it appeared that Food Science will be the first choice for most HACCP studies to be published.

**Figure 4:**
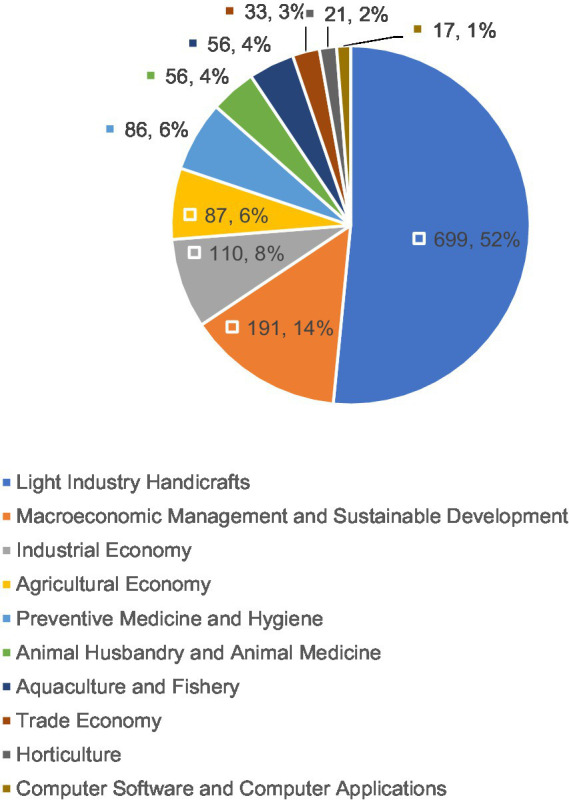
Top 10 related disciplines in the field of hazard analysis and critical control points (HACCP).

Based on Figure 4, the research disciplines in HACCP were relatively concentrated.

Figure 4 showed a relatively concentrated concentration of disciplines involved in the research and application of HACCP. These disciplines were ranked as the top 10 related disciplines: Light Industry Handicrafts, Macroeconomic Management and Sustainable Development, Industrial Economy, Agricultural Economy, Preventive Medicine and Hygiene, Animal Husbandry and Animal Medicine, Aquaculture and Fishery, Trade Economy, Horticulture, Computer Software and Computer Applications. The field of Light Industry Handicrafts accounted for the largest proportion, more than half, which indicated that the research and application of HACCP was most closely related to the discipline of Light Industry Handicrafts, followed by the discipline of Macroeconomic Management and Sustainable Development, accounting for 14%. Other subjects made up a relatively small proportion of the sample. Research conducted in the HACCP field is generally multidisciplinary, involved strong intersections, and encompassed a wide range of applications; most industries were closely related to the development of HACCP.

### 3.2. Examination of major research institutions and authors

The analysis of the main research institutions and authors in the field of HACCP research allows us to ascertain the main research forces and the levels of cooperation among the researchers. The visualization map generated by CiteSpace software consists of nodes and lines, where nodes represent major research institutions, main authors, keywords, etc. The larger the node size, the more literature that has been published by the corresponding research institution or author; connections between nodes indicate collaboration between researchers and authors. Generally speaking, the thicker the connection, the closer the cooperation will be. As opposed to this, if there are no connections between nodes, it indicates the absence of a cooperative relationship between authors and organizations.

#### 3.2.1. Main research institutions

A CiteSpace application was run with a time span of 1992–2022 set, a time slice of 1 year chosen, the node type selected as institution, and finally the data was compiled as follows: there were 498 nodes, there was one connection, there was zero network density, and the number of connections was one. It was important to note that the number of nodes represents the number of research institutions, and the connections between nodes represent some of those institutions appearing in the same literature; that was, they shared a cooperative relationship. Network density referred to the actual number of relationships divided by the maximum number of relationships in theory. It should be noted that there was no specific standard for this index. The data showed that there were many research institutions involved in HACCP, but the quantity of cross-institution collaborative research was relatively low. The main research institutions in the field of HACCP were showed in Table 1.

**Table 1:** Main research institutions in the field of hazard analysis and critical control points (HACCP).

Quantity of papers/piece	Time of first publication/year	Main research institution
32	1992	Cultivation base of State Key Laboratory of Authentic Medicinal Materials, Chinese Medicine Resource Center, China Academy of Chinese Medical Sciences
5	2014	Guangdong Province Hospital for Occupational Disease Prevention and Treatment
5	2007	School of Life Sciences, Nanchang University
5	2002	China Aquatic Product Quality Certification Center
5	2003	College of Food Science & Nutritional Engineering, China Agricultural University
4	2002	School of Food Science and Engineering, South China University of Technology
4	2004	College of Veterinary Medicine, China Agricultural University
3	2016	Harbin University of Commerce
3	2001	Shanghai Animal Husbandry and Veterinary Station
3	2001	Qingdao Exit-Entry Inspection and Quarantine Bureau
3	2007	College of Food Science, Fujian Agriculture and Forestry University
3	2006	Institute of food quality safety and testing, Jiangsu Academy of Agricultural Sciences
3	2005	School of Food Science and light Industry, South China University of Technology
3	2012	School of Environmental & Safety Engineering, Changzhou University

Based on the relevant data presented in Tables 1, a total of 14 research institutions published more than three articles related to the field of HACCP. Among them, the base of the State Key Laboratory of Authentic Medicinal Materials of Chinese Medicine Resource Center of the China Academy of Chinese Medical Sciences published the first and most articles, with a total of 32 articles in total. Guangdong Hospital for Occupational Disease Prevention and Control, the College of Life Science of Nanchang University, China Aquatic Product Quality Certification Center, and the College of Food Science and Nutritional Engineering of China Agricultural University were the other institutions with highest scientific research strength, all of which published five articles in the past year.

#### 3.2.2. Primary authors

**Figure 5:**
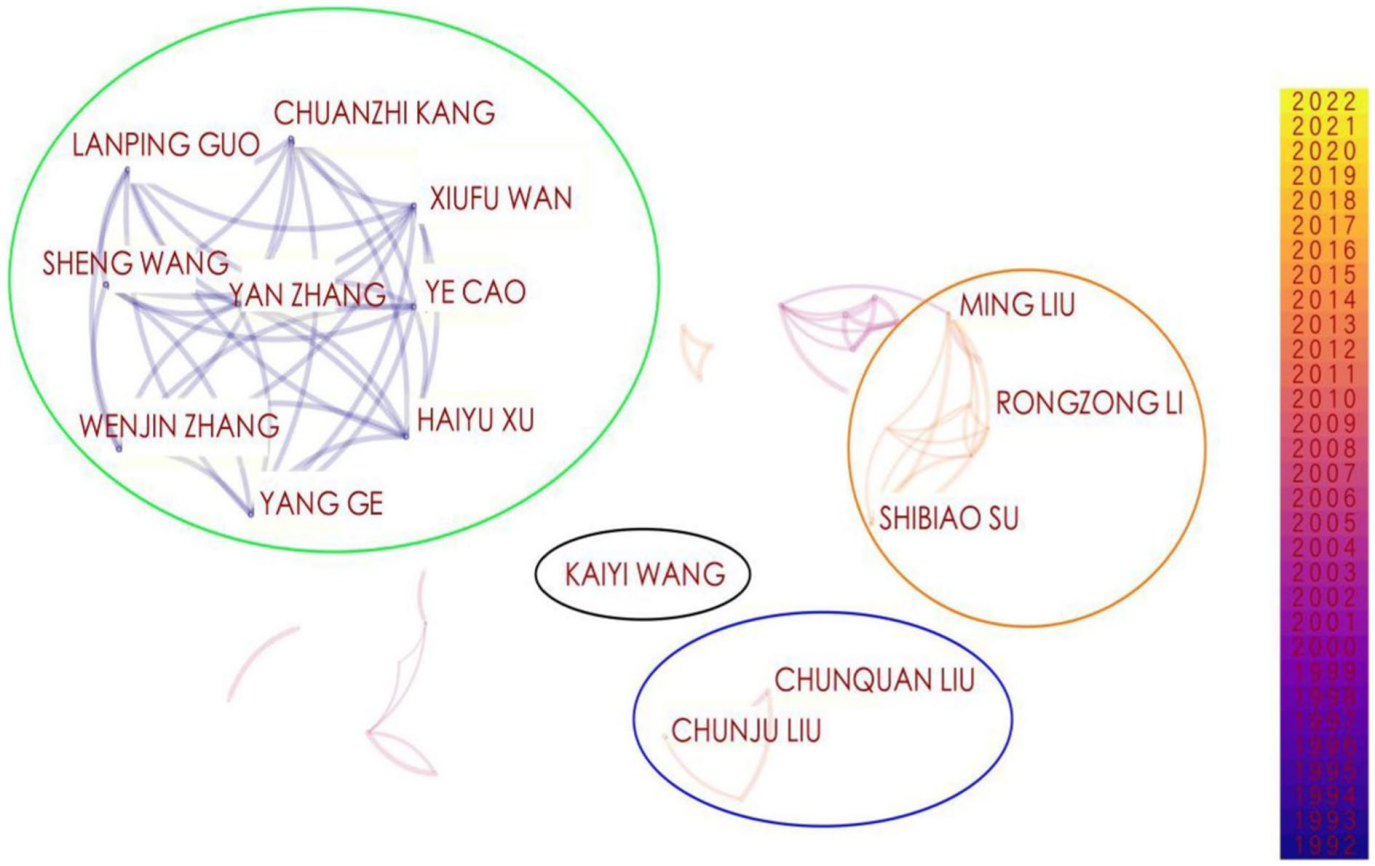
Hazard analysis and critical control points (HACCP) lead author collaboration network.

Node types in CiteSpace are set to author, and other parameters were set to the same as those in the research institution. A diagram of the lead author collaboration network in the HACCP domain could be found in Figure 5.

As can be seen from Figure 5, the main author collaboration network in the HACCP field was composed of 644 authors and 467 collaboration links, with a density of 0.0023. Based on these data, it can be seen that a large number of scholars were involved in the research area of HACCP and that there was a significant level of collaboration between researchers. For a more detailed examination of the number of papers published by the main authors in the field of HACCP and the collaboration between teams, the relevant data were summarized in Table 2.

**Table 2:** The number of articles published by primary authors in hazard analysis and critical control points (HACCP).

Number	Quantity of papers/piece	Main author
1	32	Xiufu Wan
2	19	Chuanzhi Kang
3	19	Wenjin Zhang
4	18	Yan Zhang
5	17	Ye Cao
6	13	Sheng Wang
7	12	Haiyu Xu
8	12	Yang Ge
9	11	Lanping Guo
10	9	Ming Liu
11	8	Chunquan Liu
12	6	Rongzong Li
13	6	Shibiao Su
14	5	Kaiyi Wang
15	5	Chunju Liu

The following were the main four teams related to HACCP field research, as demonstrated in Figure 5 and Table 2.

1. The research team anchored by Wan Xiufu published the largest number of papers, 32 in total, and the team members maintain frequent and close cooperation. This team primarily focuses on Chinese medicinal materials (34–38), ecological planting (39–41), ecological agriculture (42, 43).
2. The research team with Liu Ming as the core (a lot of the orange circle area) published nine articles. There was a close relationship between Liu Ming, Li Rongzong and Su Shibiao within the team, as well as a greater likelihood of cooperation between them. Liu Ming’s research team focuses mainly on the study of HACCP (44–47), occupational hazards (48–51), and risk assessment (52, 53).
3. The research team centered on Liu Chunquan (blue circle area) published five articles. In terms of the number of articles published, Liu Chunquan and Liu Chunju were the most prominent. There were three main research directions of the team: HACCP (6, 54–56), crops (57, 58), and Materia medica (59).
4. The research team led by Wang Kaiyi (black circle area) published five articles. The graph above showed Wang Kaiyi’s name separately as he published five papers related to HACCP alone. There were three major research directions emphasized by Wang Kaiyi’s team: computer software and computer applications (60, 61), agronomy (62) and agricultural economics (63, 64).

### 3.3. Analysis of research hotspots

Typically, keyword clustering is used to identify the research topics in a certain research field within a given period of time, while burst terms refer to the rapid increase or decrease in interest in a certain research field within a specific period of time. By detecting burst terms, research hotspots in different periods can be revealed (65).

#### 3.3.1. Keyword clustering

**Figure 6:**
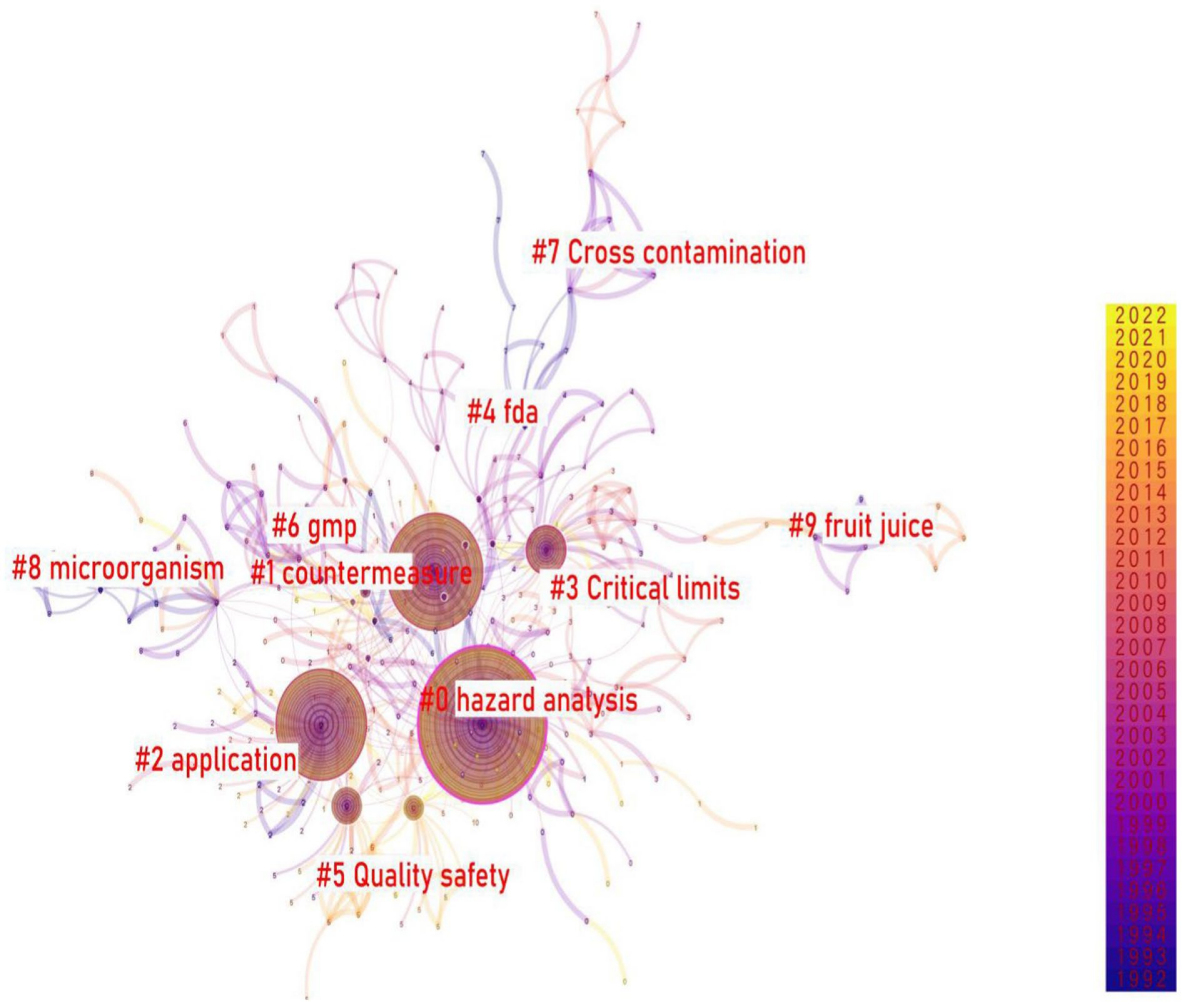
Hazard analysis and critical control points (HACCP) keyword clustering diagram.

For the purpose of in-depth exploration of the topic of HACCP research, the paper used CiteSpace software for keyword cluster analysis. Keyword cluster analysis referred to the use of clustering statistical methods based on co-occurrence analysis to simplify the occurrence network relationships into relatively small clusters. The node type was set as the keyword, and the settings of other parameters were the same as those of the research institution when the CiteSpace software is run. Based on the keyword knowledge network graph, the LLR algorithm was selected to obtain the HACCP research keyword clustering graph (Figure 6).

It can be seen from Figure 6 that the top 10 keyword clusters were hazard analysis, countermeasures, application, critical limit value, FDA, quality and safety, GMP, cross contamination, microorganism, and juice. Keyword cluster ranked from 0 to 9. The smaller the number, the more keywords were included in the cluster. A cluster consists of a number of closely related words. Which words were contained in each cluster? The keyword co-occurrence network clustering table (Table 3) was obtained by using the log-likelihood algorithm (one of the clustering label word extraction algorithms). As indicated in the table, the silhouette represents a reasonable degree of clustering. It was generally believed that silhouette 0.5 indicated reasonable clustering results; silhouette 0.7 indicated satisfactory clustering results.

**Table 3:** Clustering of keyword co-occurrence networks.

No.	silhouette	Cluster	Key words
#0	0.905	Hazard analysis	Hazard analysis; critical control point; food safety; application; application research on
#1	0.930	Countermeasures	Countermeasures; GAP; GACP; food safety; hazard analysis
#2	0.866	Application	Application; security; enterprise management; microcapsules; hazard analysis critical control point (HACCP)
#3	0.934	Critical limit value	Critical limit value; pond culture; compound feed; breeding base; pollution-free food
#4	0.956	FDA	FDA；ISO；feed industry; Canada; The management system
#5	0.938	Quality safety	Quality safety; quality control; agricultural products; vegetables; the supply chain
#6	0.915	GMP	GMP；health supervision; SSOP. Quality of hygiene; raw milk
#7	0.959	Cross contamination	Cross contamination; harm; Salmonella; safety and health; the slaughterhouse
#8	0.985	Microbes	Microbes; Canned food; system; sterilization formula; Canned food sterilization
#9	0.980	Fruit juice	Fruit juice; real time control; the key point; safe water supply; small towns

According to Table 3, the silhouettes of the top 10 clustering keywords in the HACCP field research were all greater than 0.7, which indicated that the clustering results were compelling, which also confirms the correctness of selecting HACCP as the title of this paper. For example, cluster #0, the topic of hazard analysis, mainly includes hazard analysis, critical control points, food safety, application, application research, and other keywords. These keywords were all related to the topic of food safety. The topics under the other cluster tags were generally the same as those under the first cluster tag.

According to Table 3, topics within each cluster have overlapping phenomena. In light of this, the related research in the field of Chinese HACCP can generally be categorized into two areas: “hazard analysis” and “process management.”

##### 3.3.1.1. Hazard analysis

Hazard analysis. The HACCP food safety system was a scientific and preventative system for identifying and controlling potential hazards, with the objective of minimizing the degree of harm. In hazard analysis, each link of a food production process was evaluated; the type of hazard is identified; the level of hazard was divided, the magnitude of hazard was analyzed; the significance of the hazard can be determined, critical control points were established, and a critical limit value was determined. Hazard analysis played an important role as a basis for process management, control, and prevention.

##### 3.3.1.2. Process management

Process management. Following the establishment of the critical control point, the critical limit value should be determined, and the production process should be regulated in order to improve the quality and safety of the product. Under the premise of strict control of temperature, humidity, hygiene, time, and other factors, modern production equipment should be selected as far as possible in the process of food production, and monitoring methods and frequencies should be set to monitor each link of the production process. Hazard correction measures should be determined, and the whole production process should be recorded. Food safety control and prevention work can be implemented by strictly managing the production process.

#### 3.3.2. Keyword burst

In CiteSpace software, the burst detection feature was primarily utilized to identify rapid changes in the number of references for a certain topic (66). A burst detection was viewed as an indicator of a highly active area of research that can be used to examine trends in emerging topics as well as provide a visual representation of the duration of these hotspots of research. The higher the intensity of keyword emergence, the more obvious the research orientation was, and the more it was a node of attention in a certain field of study.

**Figure 7:**
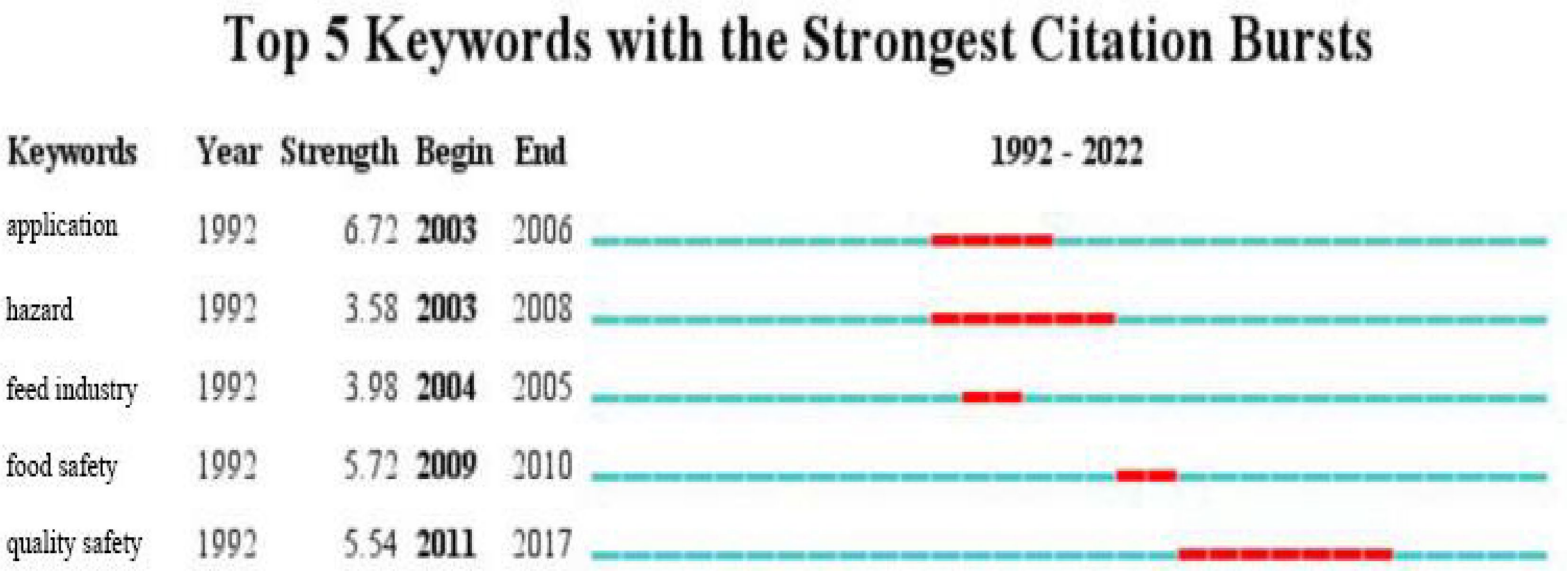
Keyword burst in the field of hazard analysis and critical control points (HACCP) research.

As shown in Figure 7, the top five keywords with burst intensity from 1992 to 2022 were identified using CiteSpace software’s burst detection function. As showed in the diagram, strength represents the intensity of the burst, begin represents the year during which bursts began, end represents the year when bursts ended, and the red part indicates the continuous burst throughout the year.

According to Figure 7, during the period 1992–2022, the keyword “application” was the highest burst intensity, with a strength of 6.72, and the start was 2003 and the end was 2006. Further, there were two keywords with a strength value greater than 5, namely “food safety” and “quality safety,” indicating that they had always been concerned, particularly for “quality safety.” Last but not least, the keyword with a strength value close to 4 was “feed industry,” which had a short emergence time and does not receive sustained attention. Although it had not been popular for a long time, the keyword “harm,” whose strength value was 3.58, was an unavoidable topic in all walks of life and had been addressed by all fields of society. Therefore, it was likely that the keyword “harm” will continue to be prominent in the future.

## 4. Discussion

Based on the analysis of the basic characteristics, main research institutions, main authors, and research hotspots in the field of HACCP in China, this paper discusses the research progress of HACCP and the research frontiers in the future. First of all, the study only counted and analyzed the number of documents issued in the HACCP field but did not count and analyze the cited quantity, which is one of the disadvantages of CiteSpace software. Later, we will continue to explore the international food safety management system. Secondly, in the future, the research focus in the field of HACCP in China will still be on hazards analysis, countermeasures, applications, quality safety, GMP, and other topics. In China, the research focus has shifted from hazard analysis to the concept of hazard analysis integrated process management, with the realization that HACCP is a strategic consideration of integrated management and is a line management from pre-production, in-process, and post-production of food. There are also challenges to be faced.

In a word, the HACCP system is an internationally recognized and accepted food safety assurance system that meets the human requirements for food safety and also improves the social requirements for food safety standards in production enterprises. The study not only improved the previous food safety management systems such as GMP, SSOP, and GAP but also promoted the “China HACCP system” to the world from experience.

## 5. Conclusion

This study used CSSCI, CSCD, and the core of Peking University as sample data sources, based on the bibliometric analysis method and CiteSpace visualization software to produce a knowledge map of research in the field of HACCP in China from 1992 to 2022, and made a systematic and detailed analysis of the basic characteristics of the literature, main research institutions and authors, and research hotspots.

### 5.1. Fundamental characteristics of the literature

Fundamental characteristics of the literature. In terms of the number of publications, there were two main trends: an upward trend between 1992 and 2004, and a downward trend between 2004 and 2022. As for the main journal sources, the journals chosen to publish research articles in the field of HACCP tend to be more concentrated, such as the journal “Food Science,” which had the most publications in the field. There was only a small proportion of articles published in other journals, and the number of articles published in each journal accounts for approximately 5% of the total number of articles published in the journal. According to this study, most HACCP researchers choose “Food Science” as their first choice. Furthermore, as far as related disciplines were concerned, the disciplines involved in the field of HACCP were also relatively specialized, among which the light industry and handicraft disciplines account for more than half of the total. This indicates that a much larger proportion of the research and applications for HACCP were focused on light industry and handicraft. Secondly, HACCP was closely related to macroeconomic management and sustainable development discipline. To sum up, HACCP field research involved multi-disciplines, strong intersections, and a wide range of applications.

### 5.2. The most important research institutions and authors

The most important research institutions and authors. As far as main research institutions were concerned, there was an abundance of research in the field of HACCP, but there was very little cross-institutional collaboration. The first and most of the papers published by the Chinese Medicine Resource Center, China Academy of Chinese Medical Sciences, are those published by the Cultivation base of the State Key Laboratory of Authentic Medicinal Materials. In terms of main authors, four active research teams had been formed in the field of HACCP, with Wan Xiufu, Liu Ming, Liu Chunquan, and Wang Kaiyi as the core, respectively. The main research directions included HACCP, Chinese medicine, ecological planting, ecological agriculture, occupational hazards, risk assessment, crops, computer software and computer applications, agronomy, agricultural economy, etc. The authors on each team collaborated closely.

### 5.3. Research hotspots

Research hotspots. As for keyword clustering, if the silhouette was greater than 0.7 after screening keywords related to HACCP field research, this indicated that the clustering results were satisfactory, ensuring the correctness of using HACCP as the title of the article. Further, there were overlapping phenomena among the research topics under each cluster, and related research in the Chinese HACCP field can be categorized into two areas: “hazard analysis” and “process management.” From 1992 to 2022, the keyword “application” had the highest burst intensity, followed by “food safety” and “quality safety,” indicating that these three topics had always attracted attention. In spite of the fact that the keyword “hazard” had not burst for a long time, the research related to this topic has always attracted the attention of all areas of society, and it had become an unavoidable topic in all walks of life. Therefore, the burst may continue to come into play for a longer period of time.

## Author contributions

XS, XZ: conceptualization, data curation, and writing—original draft, XS, XZ, RA,TW, JZ, and YL: formal analysis, XS: funding acquisition, methodology; XS, TW: supervision, XS, XZ, and RA: writing—review and editing. All authors contributed to the article and approved the submitted version.

## Funding

This work was supported by the Thermal Sterilization Process on the Characteristic Flavor Substances of Canned Chestnut, grant number 21327117D, the Fund for Education Department of Liaoning Province, grant number WSNJC202035, the Fund for Postdoctoral Research of Shenyang Agricultural University, grant number 770218007, the introductory Talent Research Start Project of Shenyang Agricultural University, grant number 2016, the Horizontal project of Fudan University, grant number H2022040, the Horizontal project of Beijing Zhongji Huada Technology Development Co., Ltd., grant number H2022121, and the National Fund Cultivation Project, School of Economics and Management, Shenyang Agricultural University, grant number JGPY20170203.

## Conflict of interest

The authors declare that the research was conducted in the absence of any commercial or financial relationships that could be construed as a potential conflict of interest.

## Publisher’s note

All claims expressed in this article are solely those of the authors and do not necessarily represent those of their affiliated organizations, or those of the publisher, the editors and the reviewers. Any product that may be evaluated in this article, or claim that may be made by its manufacturer, is not guaranteed or endorsed by the publisher.

## Footnotes

^1^ http://cluster.ischool.drexel.edu/-cchen/CiteSpace/download/
